# Transthyretin Amyloidosis Due to a Rare TTR Val142del Deletion With Relatively Early Clinical Manifestations

**DOI:** 10.1016/j.jaccas.2026.108772

**Published:** 2026-09-23

**Authors:** Cristhian Espinoza Romero, Kevin De Paula Morales, Christiane Espirito Santo, Guilherme dos Santos Ferreira, Alan Silva Martins, Bruno Vaz Kerges Bueno, Fabio Fernandes

**Affiliations:** Instituto do Coração (InCor), Hospital das Clínicas HCFMUSP, Faculdade de Medicina, São Paulo, Brazil

**Keywords:** amyloidosis, autonomic nervous system diseases, familial, genetic screening, mutation, transthyretin

## Abstract

**Background:**

Hereditary transthyretin amyloidosis shows marked phenotypic heterogeneity. In-frame deletions at codon 142 are exceedingly rare, and their cardiac expression remains poorly characterized.

**Case Summary:**

A 62-year-old man presented with progressive neuropathy, autonomic dysfunction, weight loss, and recurrent syncope. Multimodality imaging demonstrated concentric hypertrophy with preserved ejection fraction, apical sparing on strain, diffuse late gadolinium enhancement with increased extracellular volume, and grade III uptake on 99mTc-pyrophosphate scintigraphy. Monoclonal gammopathy was excluded. Genetic testing identified a heterozygous the transthyretin p.Val142del deletion. Autonomic testing confirmed orthostatic hypotension with impaired chronotropic response. Heart failure therapy and tafamidis led to symptomatic improvement. Cascade screening identified 2 asymptomatic carriers.

**Discussion:**

Unlike the common Val142Ile substitution, this deletion appears associated with earlier penetrance and multisystem involvement.

**Take-Home Messages:**

Rare transthyretin deletions may cause aggressive hereditary transthyretin amyloidosis. Early recognition and family screening are essential.

## Graphical Abstract

**Figure:**
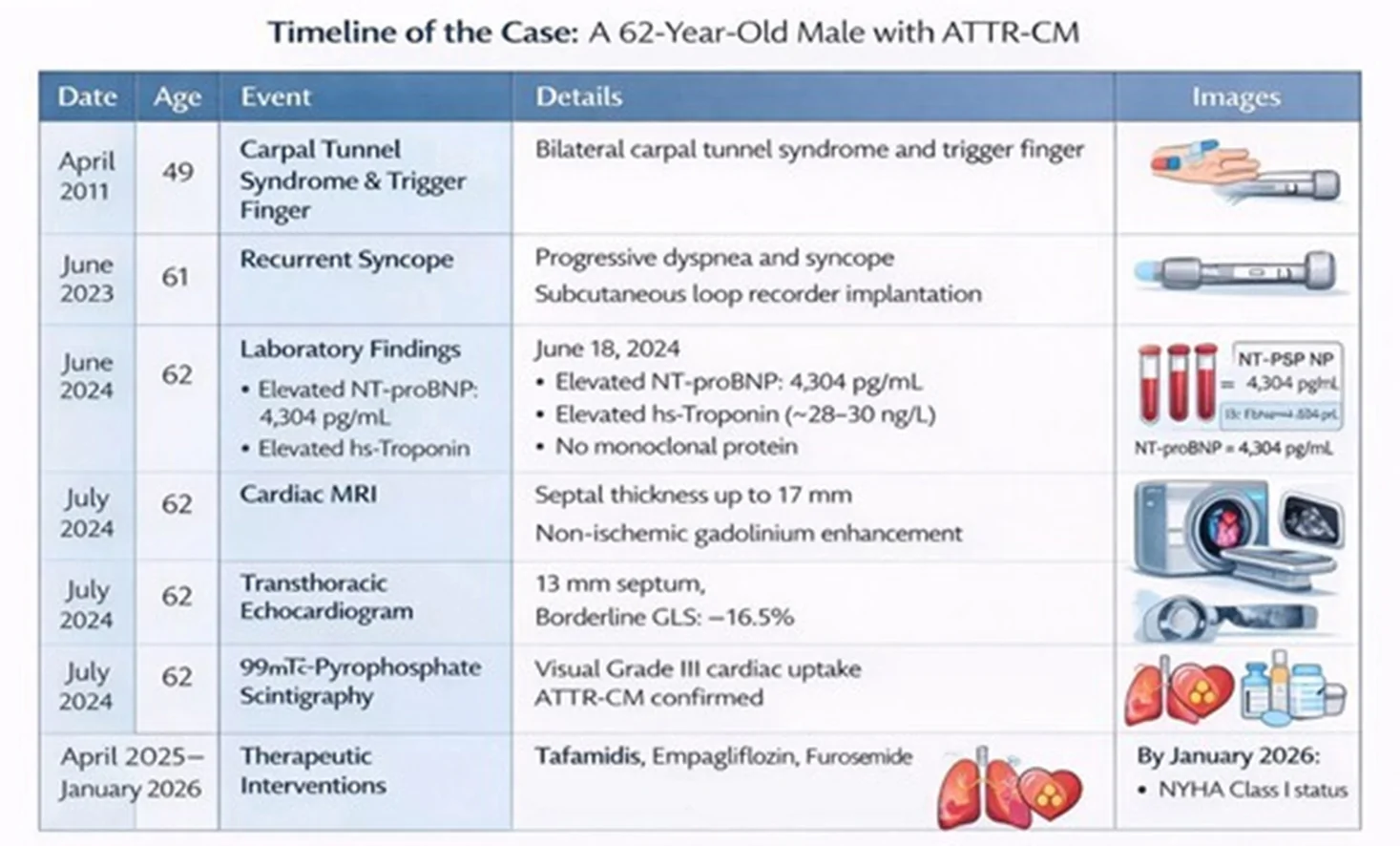

## History of Presentation

**Figure 1:**
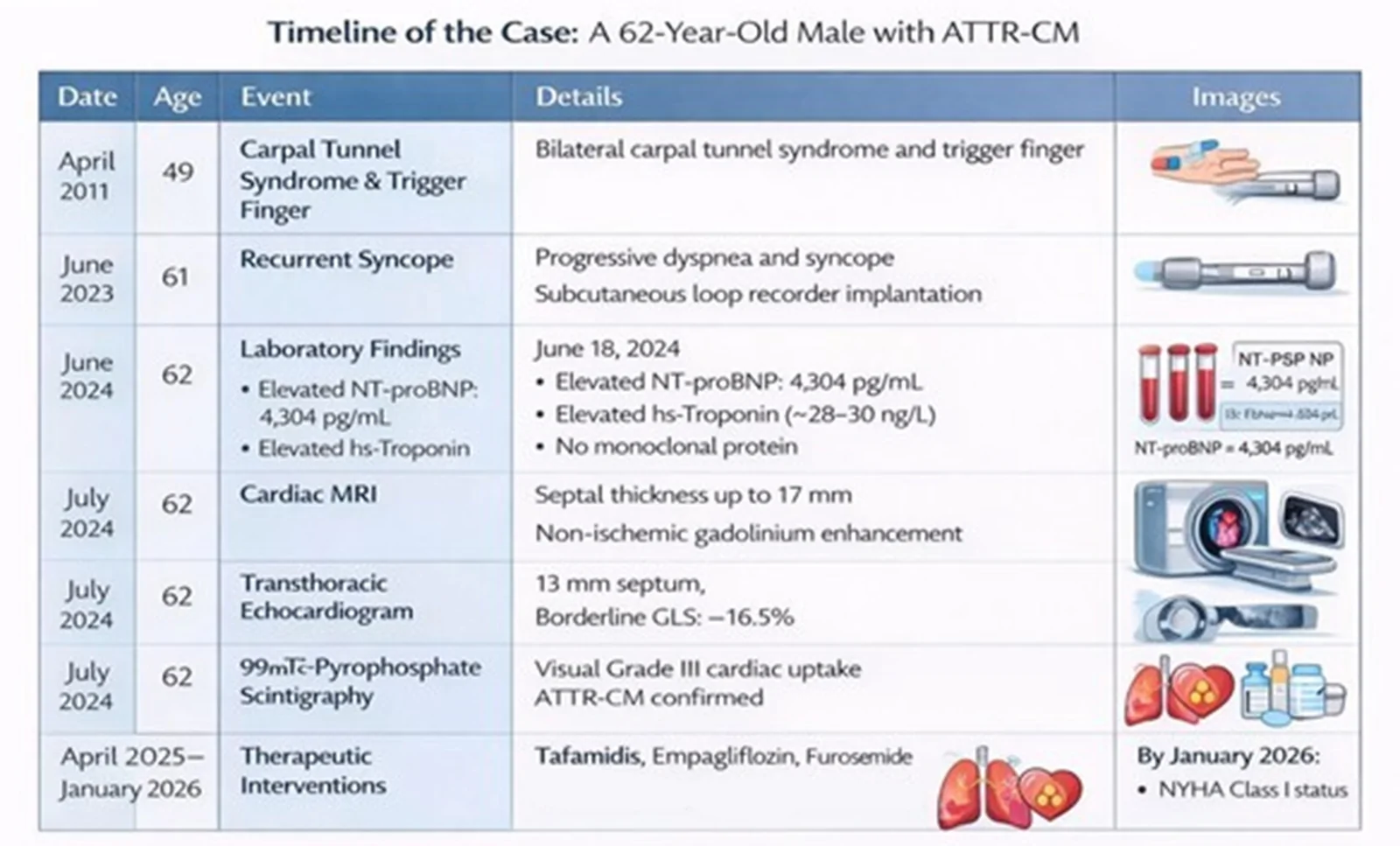
Timeline of the Clinical Course Chronological overview of key clinical events, diagnostic findings, multimodality cardiac imaging, and therapeutic interventions in a patient with hereditary transthyretin cardiac amyloidosis associated with the transthyretin Val142del variant. ATTR-CM = transthyretin cardiac amyloidosis; GLS = global longitudinal strain; hs-troponin = high-sensitivity troponin; MRI = magnetic resonance imaging; NT-proBNP = N-terminal pro–B-type natriuretic peptide.

A 62-year-old male physician presented with progressive systemic and cardiovascular symptoms over 18 months. He reported recurrent syncope associated with orthostatic intolerance, exertional dyspnea, progressive distal sensory neuropathy, autonomic dysfunction (urinary and sexual dysfunction), intermittent dysphagia, and unintentional 5-kg weight loss (Figure 1).

**Take-Home Messages**

• Rare in-frame deletions in the TTR gene, including p.Val142del, may present with relatively early clinical manifestations of multisystem hereditary transthyretin cardiac amyloidosis and significant cardiac involvement detectable using established noninvasive diagnostic criteria.
• Early genetic testing, multimodality imaging, and cascade family screening are critical to enable timely initiation of disease-modifying therapy.

On physical examination, blood pressure was stable in the supine position, with symptoms reproduced on orthostasis. Mild bibasilar crackles were present without peripheral edema. Cardiac auscultation was unremarkable.

## Past Medical History

The patient had no history of hypertension, diabetes mellitus, smoking, or alcohol use.

Notably, he underwent bilateral carpal tunnel release and trigger finger surgery approximately 12 years before the diagnosis, with recurrence of trigger finger occurring 1 year before diagnosis. There was no previously known family history of cardiomyopathy or neuropathy.

## Investigations

Laboratory evaluation revealed persistently elevated high-sensitivity troponin (28-30 ng/L) and N-terminal pro–B-type natriuretic peptide of 4,304 pg/mL, with preserved renal function. Serum and urine immunofixation, free light-chain assay, bone marrow biopsy, and abdominal fat pad biopsy excluded monoclonal gammopathy.

**Figure 2:**
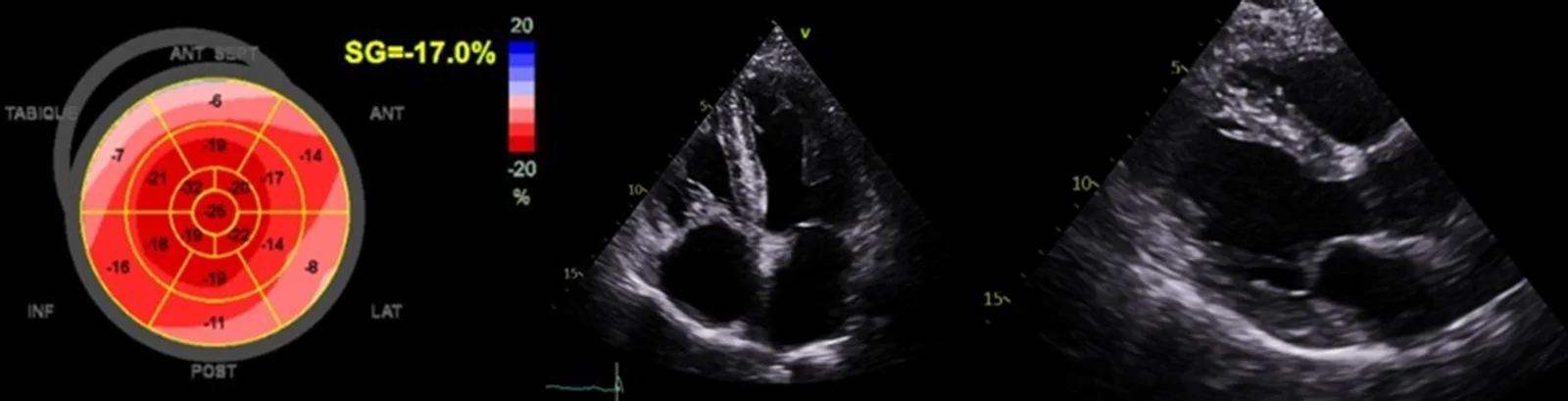
Transthoracic Echocardiography and Strain Imaging Apical 4-chamber and parasternal long-axis views demonstrating mild concentric left ventricular hypertrophy and preserved systolic function, with global longitudinal strain showing relative apical sparing.

Electrocardiography demonstrated sinus rhythm, right bundle branch block, low voltage in limb leads, and diffuse repolarization abnormalities. Transthoracic echocardiography showed mild concentric left ventricular hypertrophy (septal and posterior wall thickness 13 mm), preserved ejection fraction (65%), and relative apical sparing on global longitudinal strain (−17%) (Figure 2).

**Figure 3:**
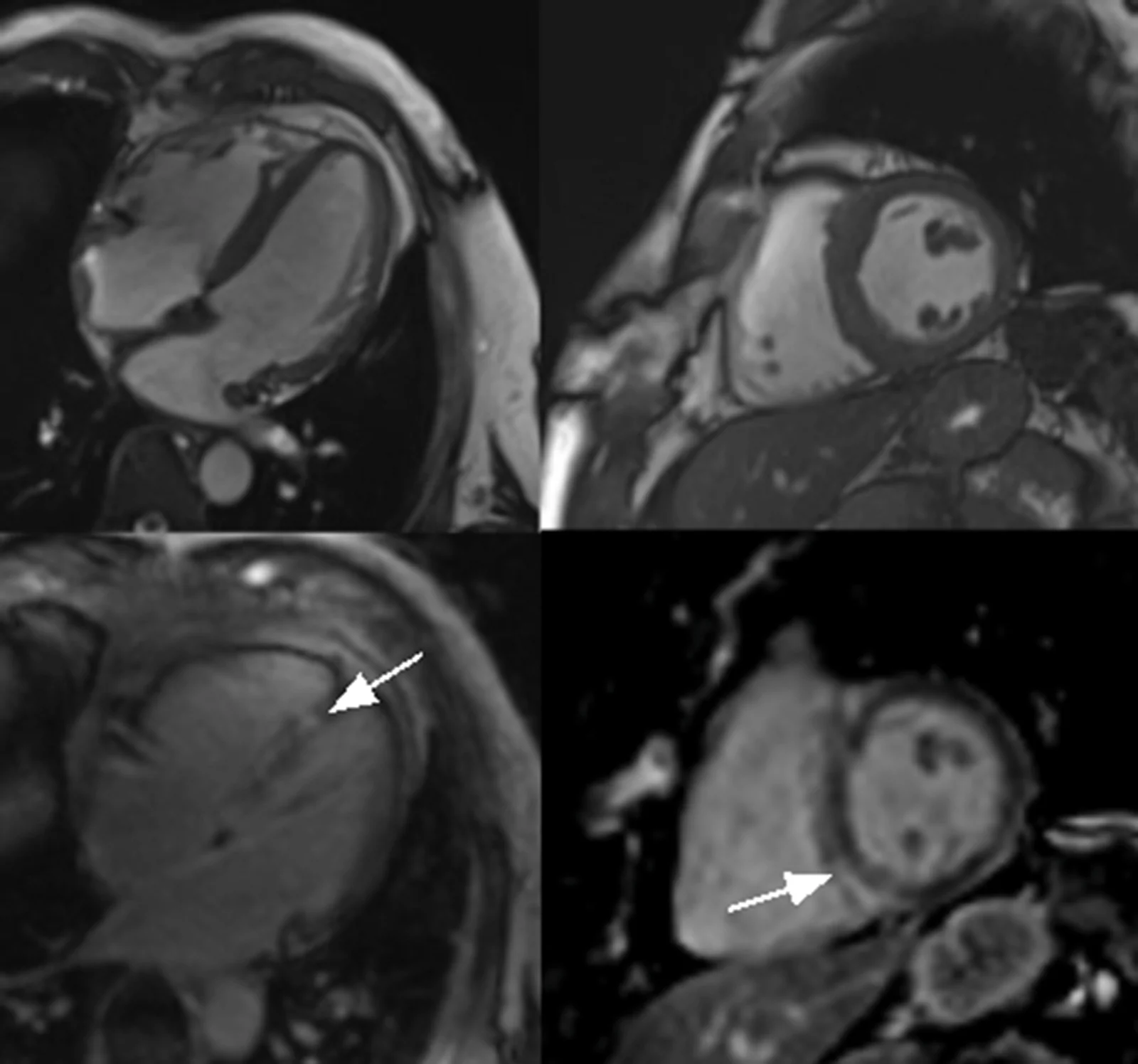
Cardiac Magnetic Resonance Imaging Cine imaging and late gadolinium enhancement sequences demonstrate increased interventricular septal thickness and diffuse, patchy, nonischemic myocardial enhancement, predominantly involving the intramyocardial and septal regions (white arrows), consistent with cardiac amyloidosis.

**Figure 4:**
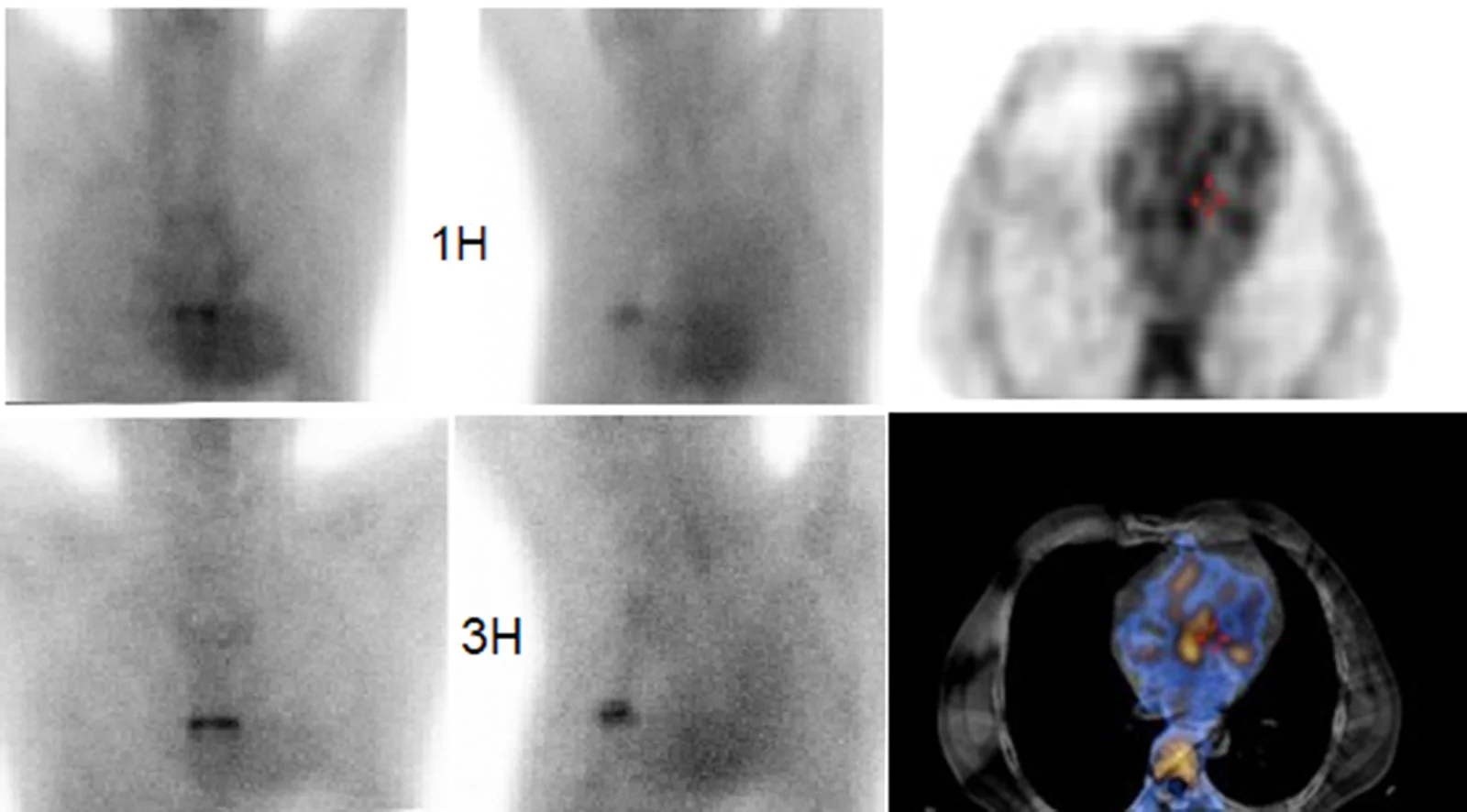
99mTc-Pyrophosphate Scintigraphy Intense grade III myocardial uptake exceeding rib uptake, diagnostic of transthyretin cardiac amyloidosis. 1H = first hour; 3H = third hour.

Cardiac magnetic resonance revealed asymmetric septal hypertrophy (maximal thickness 17 mm), diffuse nonischemic late gadolinium enhancement, prolonged native T1, and markedly increased extracellular volume (45%), consistent with infiltrative cardiomyopathy (Figure 3). 99mTc-pyrophosphate scintigraphy demonstrated grade III myocardial uptake greater than rib uptake, establishing transthyretin cardiac amyloidosis (ATTR-CM) in the absence of monoclonal protein (Figure 4, Video 1).

Nerve conduction studies and electromyography were performed and revealed severe bilateral median neuropathy at the wrist, consistent with advanced carpal tunnel syndrome, in addition to a chronic, symmetric, length-dependent sensorimotor polyneuropathy with predominantly axonal features and evidence of chronic reinnervation in distal muscles. These findings support the presence of systemic peripheral nerve involvement.

**Figure 5:**
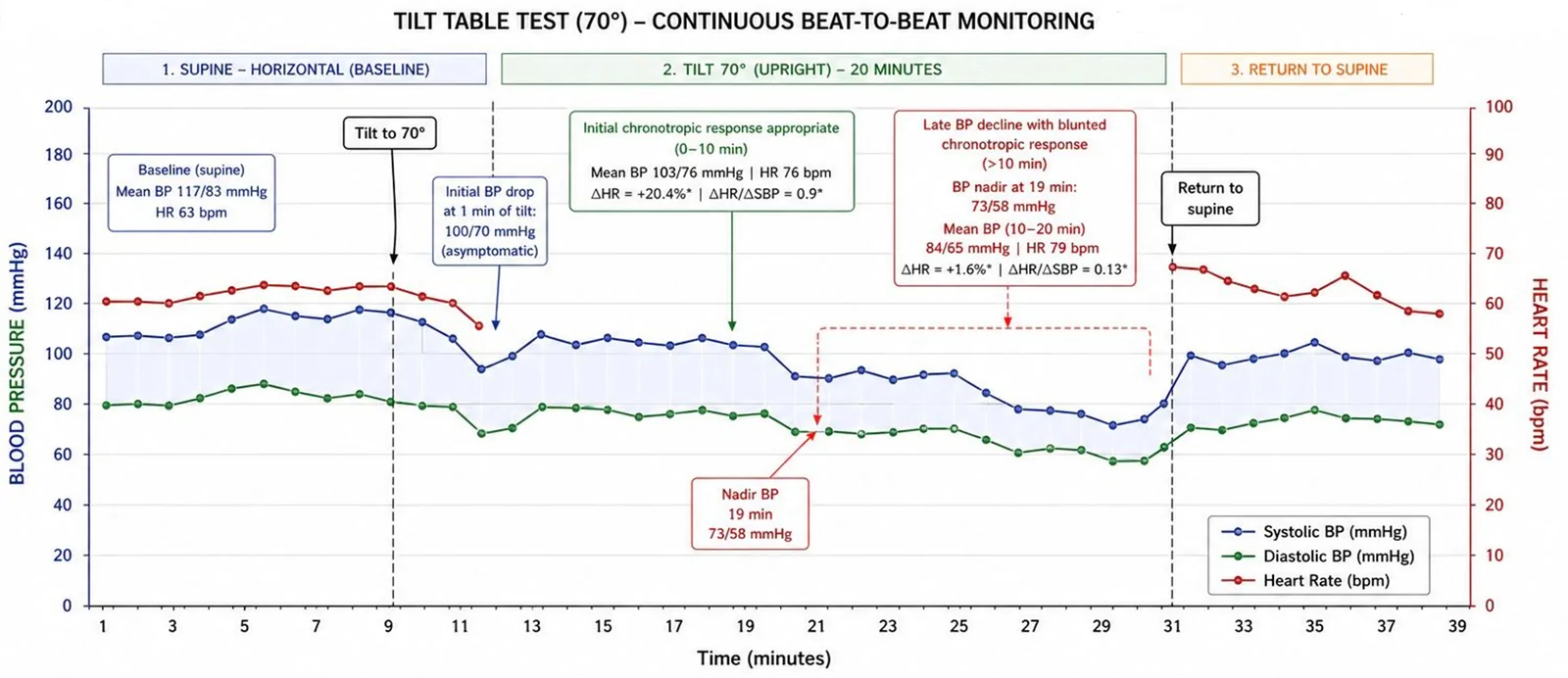
Head-Up Tilt-Table Test Beat-to-beat systolic blood pressure (SBP) (blue) and diastolic (green) blood pressure (BP) and heart rate (HR) (red) during supine rest and 70° tilt. An initial BP decrease with appropriate HR increase is followed by a progressive late BP decline (>10 minutes) with inadequate chronotropic response, consistent with dysautonomic orthostatic hypotension. ΔHR = heart rate change; ΔHR/ΔSBP = ratio of heart rate to systolic blood pressure variation.

Head-up tilt testing confirmed classical and delayed orthostatic hypotension with impaired chronotropic response (Δheart rate/Δsystolic blood pressure = 0.13), consistent with autonomic dysfunction (Figure 5). Genetic testing identified a heterozygous in-frame deletion in transthyretin (TTR) (c.424_426delGTC; p.Val142del), classified as pathogenic.

## Differential Diagnosis

Given the multisystem involvement and cardiac findings, the differential diagnosis included the following:

Light-chain amyloidosis was the primary alternative diagnosis; however, it was excluded by negative serum and urine immunofixation, normal free light-chain assay, and negative bone marrow and fat pad biopsies.

Wild-type transthyretin amyloidosis was considered unlikely because of the patient's age, prominent neuropathic and autonomic features, and subsequent identification of a pathogenic TTR variant.

Hypertensive heart disease was excluded in the absence of a history of hypertension and given the presence of low-voltage electrocardiography and characteristic apical sparing on global longitudinal strain imaging.

Hypertrophic cardiomyopathy was considered; however, the diffuse late gadolinium enhancement pattern, markedly elevated extracellular volume, and positive 99mTc-pyrophosphate scintigraphy favored ATTR-CM.

Fabry disease was also considered given the presence of cardiac and neuropathic involvement; however, it was deemed unlikely because of the absence of typical extracardiac manifestations (such as angiokeratomas, corneal verticillata, renal dysfunction, or proteinuria) and the imaging and scintigraphic findings consistent with ATTR-CM.

Cardiac sarcoidosis was deemed unlikely because of the absence of systemic inflammatory features and the scintigraphic findings diagnostic of ATTR-CM.

The constellation of findings supported a diagnosis of hereditary transthyretin cardiac amyloidosis.

## Management

The patient was classified as National Amyloidosis Centre stage II and Mayo stage II ATTR-CM. Heart failure therapy was initiated with diuretics, eplerenone 25 mg once daily and empagliflozin 10 mg once daily, resulting in partial symptomatic improvement. Tafamidis 20 mg orally once daily therapy was subsequently introduced as disease-modifying treatment. Close multidisciplinary follow-up was implemented, including cardiology and neurology assessment. Cascade genetic screening of first-degree relatives was performed.

## Outcome and Follow-Up

Following initiation of tafamidis and optimized heart failure therapy, the patient demonstrated progressive clinical improvement. By January 2026, he was asymptomatic from a cardiovascular standpoint (NYHA functional class I), with good treatment tolerance. This was accompanied by a marked reduction in N-terminal pro–B-type natriuretic peptide levels from 4,304 pg/mL at baseline to 330 pg/mL, while high-sensitivity troponin remained stable (28-30 ng/L at baseline vs 32.1 ng/L at follow-up). Cascade screening identified 2 asymptomatic sons (aged 27 and 29 years) heterozygous for the same TTR p.Val142del variant. The older son underwent comprehensive evaluation without evidence of cardiac or neurological involvement at the time of assessment.

## Discussion

Hereditary transthyretin cardiac amyloidosis is an autosomal-dominant systemic disorder caused by pathogenic variants in the TTR gene, leading to tetramer destabilization, monomer dissociation, and extracellular amyloid deposition in peripheral nerves and myocardium.^1^ More than 140 pathogenic variants have been described, resulting in substantial heterogeneity in age at onset, organ involvement, and disease trajectory.^2^

According to contemporary diagnostic frameworks and expert consensus documents, ATTR-CM can be established noninvasively when grade II-III myocardial uptake on bone-avid tracer scintigraphy is present in the absence of monoclonal protein.^3^ Our patient fulfilled these criteria, demonstrating grade III 99mTc-pyrophosphate uptake and negative monoclonal studies, thereby meeting consensus-supported diagnostic standards.

The Val142Ile (Val122Ile) substitution is the most prevalent cardiomyopathy-associated TTR variant worldwide and is classically associated with late-onset restrictive cardiomyopathy, typically after the sixth decade, with limited neurological involvement.^4^ In contrast, in-frame deletion variants affecting codon 142 are exceedingly rare. The p.Val142del mutation results in deletion of a single valine residue without altering the reading frame and represents one of the few structural TTR variants described.^5^ Structural studies suggest that deletion mutations may induce greater conformational instability than single amino acid substitutions, thereby increasing amyloidogenic potential.^5^

A prior report by Uemichi et al^6^ described an Ecuadorian heterozygous carrier presenting with neuropathy and autonomic dysfunction in his early 60s, with limited cardiac characterization.^6^ Our case expands the clinical spectrum by providing systematic multimodality cardiac imaging, objective documentation of autonomic dysfunction, and longitudinal follow-up under disease-modifying therapy.

Current therapeutic recommendations support early initiation of TTR stabilizers in symptomatic ATTR-CM to reduce mortality and cardiovascular hospitalizations.^3^ Notably, although rare structural variants such as Glu51–Ser52 duplication have been associated with aggressive disease and reduced response to stabilizers,^7^ our patient demonstrated favorable clinical evolution after tafamidis initiation, suggesting that tetramer stabilization remains an effective strategy in this deletion variant.

The apparent clustering of Val142del in Ecuadorian families underscores the importance of genetic testing and cascade screening in at-risk populations, as recommended by contemporary consensus statements.^3^ Early red flags, including bilateral carpal tunnel syndrome years before cardiac diagnosis, reinforce the need for heightened clinical suspicion. Given the limited data on penetrance and natural history of rare TTR deletions, systematic family screening and longitudinal phenotyping are essential to clarify genotype–phenotype correlations and optimize individualized management. Disease-modifying therapies currently include TTR stabilizers, such as tafamidis, and gene-silencing agents, such as vutrisiran, both of which have demonstrated benefit in slowing disease progression. Although gene-silencing therapy would also represent an appropriate strategy for this mixed-phenotype patient, tafamidis was selected because of greater accessibility within our health care system.

## Conclusions

This case expands the phenotypic characterization of the rare TTR p.Val142del variant, demonstrating relatively early clinical penetrance, multisystem involvement, and definitive cardiac expression established according to contemporary noninvasive diagnostic criteria. Despite its potential structural instability, treatment with tafamidis was associated with favorable clinical evolution. Rare deletion variants warrant heightened awareness, systematic genetic evaluation, and early therapeutic intervention.

### Patient Consent

The authors confirm that written informed consent for publication of clinical details and images was obtained from the patient in accordance with institutional policy, HIPAA requirements, and applicable regulations. All identifying information has been appropriately anonymized. Institutional review board approval was not required for this case report per journal guidelines.

## Funding Support and Author Disclosures

The authors have reported that they have no relationships relevant to the contents of this paper to disclose.

## Appendix

Video 1 Short-Axis Cine Imaging Demonstrating Preserved Ejection Fraction and Increased Wall Thickness, Most Evident in the Interventricular Septum
